# Supplementary material for: Single-molecule localization microscopy reveals the ultrastructural constitution of distal appendages in expanded mammalian centrioles
Source: Nat Commun. 2023 Mar 27;14:1688. doi: 10.1038/s41467-023-37342-x (PMC10043031; doi:10.1038/s41467-023-37342-x)
Supplement: Supplementary file 1 — Supplementary Information [file 41467_2023_37342_MOESM1_ESM.pdf]

Supplementary Information

Single-molecule localization microscopy reveals the ultrastructural constitution of distal appendages in expanded mammalian centrioles

Ting-Jui Ben Chang[1,2,3], Jimmy Ching-Cheng Hsu[3], T. Tony Yang*[3,4]

[1]Department of Physics, National Taiwan University, Taipei, 10617, Taiwan

[2]Nano Science and Technology Program, Taiwan International Graduate Program, Academia Sinica and National Taiwan University, Taipei, 10617, Taiwan

[3]Department of Electrical Engineering, National Taiwan University, Taipei, 10617, Taiwan

[4]Graduate Institute of Biomedical Electronics and Bioinformatics, National Taiwan University, Taipei, 10617, Taiwan

*Corresponding author:

T. Tony Yang

tonyyang@ntu.edu.tw

Supplementary Note 1. Optimization of expansion factor in the re-embedding process.

To achieve a higher expansion factor after re-embedding, we take two conditions into account – with or without Tris buffer and with or without bind-silane coating. The 2-cm hydrogel was re-embedded with either commonly-used bind-silane coating or without and compared in terms of its length changed in two solutions (**Supplementary Figure 2**). We first prepared two solutions, and both of these contained 10% acrylamide, 0.15% N,N'-methylenebisacrylamide, 0.05% TEMED, and 0.05% APS; here, the only difference was in the addition of either Tris buffer (in Solution-1, **Supplementary Figure 2a** and **b**) or ddH_2O (in Solution-2, **Supplementary Figure 2a** and **b**). Our result indicated that the re-embedding process without Tris buffer could enhance the retention rate by ~3% in one dimension compared with the process with Tris buffer (**Supplementary Figure 2c**). In the identical process of post re-embedding to rehydration in PBS, the length of the hydrogel free of bind-silane binding increases by 11% (from 1.66 cm to 1.84 cm), compared to that with bind-silane binding, valid for either re-embedding solutions (**Supplementary Figure 2c**). By direct comparison of the two solutions as well as the coating methods, we found two factors that make our re-embedded hydrogel achieve a higher expansion factor than the other work by overall enhancement of at least ~14% (from 1.66 cm to 1.89 cm).

Supplementary Note 2. Workflow of in-situ drift correction.

The practical in-situ drift correction performed in this work is illustrated in **Supplementary Figure 3**. In **Supplementary Figure 3a**, we provide the images of both the in-situ maker (ATP synthase stained with AF488) and target protein (Ac-Tub labeled with AF647) in its corresponding channels for clarity. The region of interest (ROI) for correction and Ex-dSTORM imaging were marked respectively for the following processing.

The image acquisition process is composed of incessant imaging channel excitation (637 or 561 nm laser) with intermittent 488-nm laser firing (**Supplementary Figure 3b**). It becomes convenient to discern the on-and-off states of in-situ markers by measuring intensity in correction ROI, as shown in **Supplementary Figure 3b**, where each burst is due to 488-nm excitation. From here, the lateral drift against time was plotted according to the correlation of in-situ markers across all frames taken in the correction ROI (**Supplementary Figure 3b**). The imaging series in the target ROI was then corrected and processed with its corresponding in-situ drift correction result. Sample image

pair of the corrected and uncorrected condition is shown, and a special remark is given to the ambiguity of molecular structure displayed in the uncorrected image and the enhancement of detail shown in the corrected image (**Supplementary Figure 3c**). Exceptionally, our target centriolar proteins (Ac-Tub) can be preserved with molecular details where iconic triplets are resolved following in situ drift correction. Although we could identify the nine cluster signals from the uncorrected image, the microtubule triplet arrangement could not be revealed. After in-situ drift correction, we could unravel the arrangements of microtubule triplets as marked with dashed squares and arrowheads.

Supplementary Note 3. Intensity asymmetry of C2CD3.

In order to resolve the discrepancy between the observed nine-fold symmetry of C2CD3 in our images and the asymmetric pattern reported in the previous wor[1], we scrutinized all our results of C2CD3 in Ex-dSTORM imaging and its corresponding ExM imaging. Here, two interesting patterns of C2CD3 could be categorized– full ring and intensity asymmetry (**Supplementary Figure 6**). Special note is given to the images of the intensity asymmetry part. In our ExM images, we observed asymmetric labeling as that mentioned in the previous paper. However, in the corresponding Ex-dSTROM images, we could still observe the nine-fold symmetric pattern of C2CD3 with some comparatively weak clusters. This phenomenon could be further disclosed in the saturated Ex-dSTORM images. Typically, in dSTORM, the optical setup couples with the electron multiplication charge-coupled devices (EMCCD) to detect low-intensity light sources. Therefore, dSTORM stands as an excellent imaging tool that allows single-molecule detection. Even in a seemingly incomplete ring, molecules in the depletion region can still be revealed with low localization intensity. Based on this, we deemed that the asymmetry labeling of C2CD3 previously proposed is more likely due to some varying amount of localization within nine C2CD3 puncta than the missing nine-fold symmetric distribution.

a

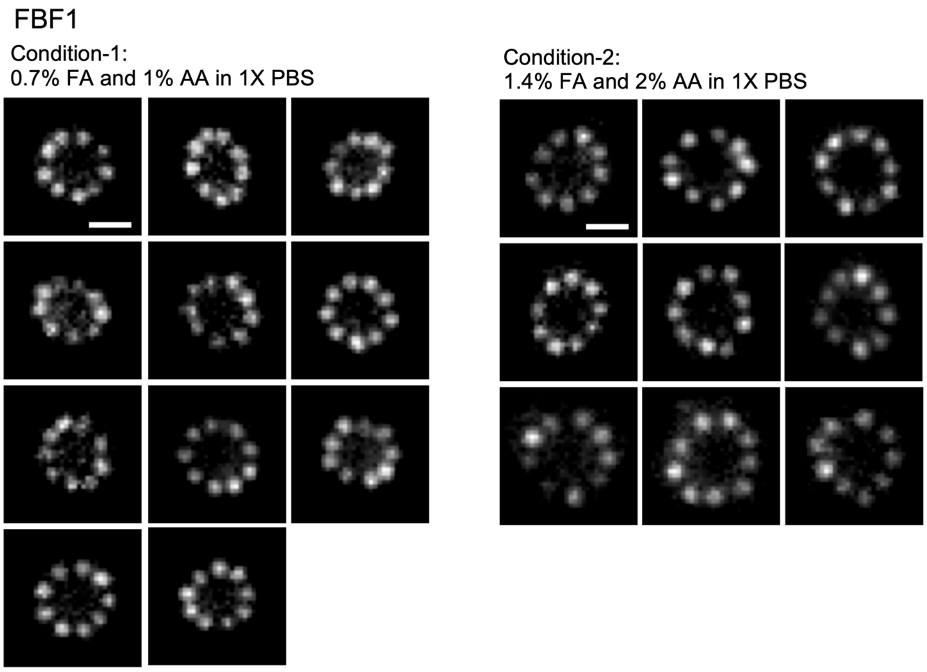

b

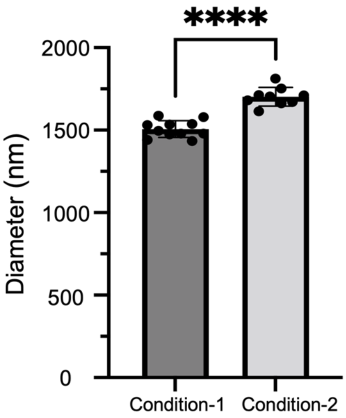

c

Diameter of FBF1

	Mean diameter (nm)	Expansion factor
Condition-1	1506 ± 51 (n=11)	3.51
Condition-2	1702 ± 57 (n=9)	3.97
Rate of increase (volume)	45 %	

Supplementary Figure 1. Optimization for the perfusion step to enhance the expansion factor. a ExM images of FBF1 under two perfusion conditions. Condition-2 doubles FA and AA concentration compared with the original recipe (condition-1), which achieves a higher expansion factor. **b** Statistical analysis with the significant difference of mean diameter under two conditions (mean ± SD, n = 11 and 9 cells for Condition-1 and Condition-2, respetively). ****p<0.0001 (p = 0.00000019), unpaired two-tailed t-test. **c** Table of mean diameter under two conditions with corresponding expansion factors estimated with our previous dSTORM result of mean FBF1 diameter[2]. Scale bar, 1 μm (**a**). Source data are provided as a Source Data file.

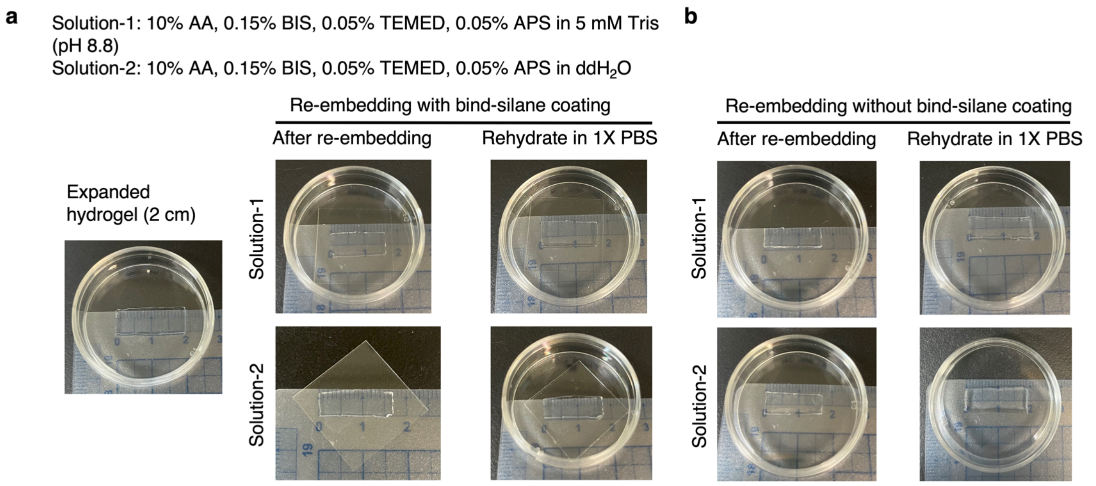

c

	Re-embedding with bind-silane coating		Re-embedding without bind-silane coating	
	After re-embedding	Rehydrate in 1X PBS	After re-embedding	Rehydrate in 1X PBS
Solution-1	1.62 ± 0.028 (81%)	1.66 ± 0.021 (83%)	1.65 ± 0.014 (82.5%)	1.84 ± 0.014 (92%)
Solution-2	1.69 ± 0.007 (84.5%)	1.72 ± 0.014 (86%)	1.70 ± 0.007 (85%)	1.89 ± 0.014 (94.5%)

Unit: cm (retention rate)

Supplementary Figure 2. Comparison of different re-embedding processes. a,b Two solutions listed on the top were prepared for re-embedding processes, and expanded hydrogels were trimmed to the size of 2 cm in length. The images of the gel were taken following re-embedding and rehydration processes in two solution conditions with bind-silane coating or without the coating, **a** for two re-embedding processes with bind-silane coating and **b** for the processes without the bind-silane coating. **c** Size of the hydrogel in each condition from **a** and **b** was measured and quantitatively analyzed with the introduction of retention rate, which is defined by the length measured divided by the original length of hydrogels (2 cm) prior to re-embedding (n = 2 independent experiments for each condition). The blue-labeled result marks the condition of the previous report; the red-labeled result indicates the condition of our work. Source data are provided as a Source Data file.

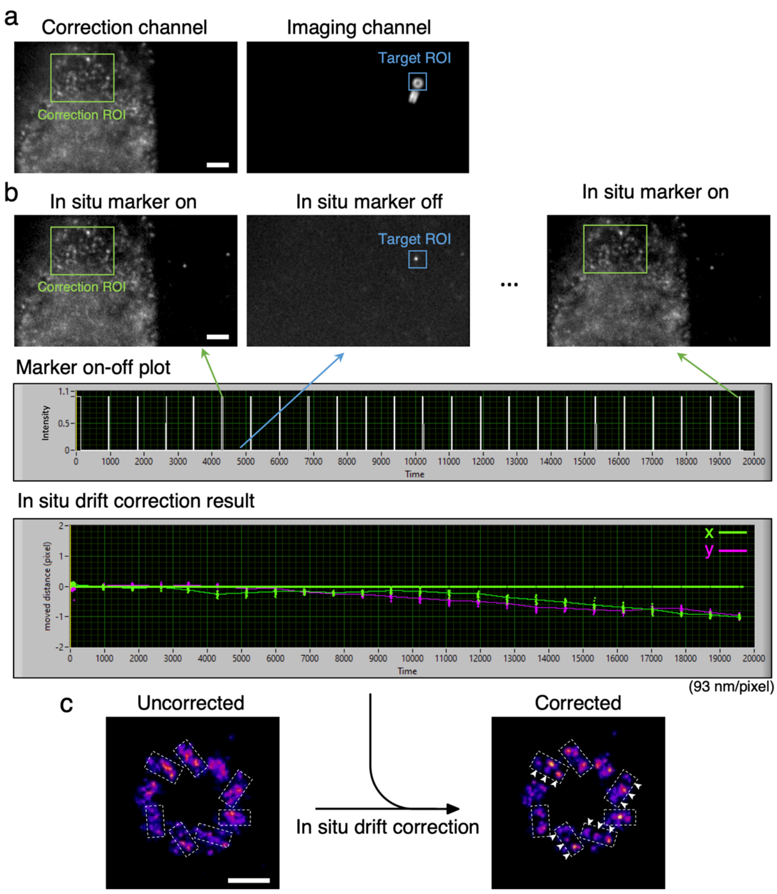

Supplementary Figure 3. Workflow of in-situ drift correction. a Representative images of in-situ marker and target protein in the correction channel (488-nm laser excitation) and imaging channel (637- or 561-nm laser excitation), respectively. Green and blue boxes represent the regions of interest (ROI) for in-situ drift correction and target imaging range. The in-situ correction will be processed in the imaging channel. Here we provide the image of an in-situ marker for clarity. **b** During the image acquisition process, the in-situ marker (stained with AF488) was intermittently illuminated (for every 800 frames) in the imaging channel. The corresponding images of single-molecule blinking with the on or off state of the in-situ marker were shown. Following the on-and-off switching of in-situ markers, the lateral drift of in-situ markers was obtained by tracking the location of markers, shown in the in-situ drift correction result. **c** Comparison of uncorrected and corrected Ex-dSTORM images. The corrected image is obtained by post-processing position compensation according to the in-situ drift correction result. The target proteins (Ac-Tub) are preserved with molecular details where iconic triplets are resolved (marked with dashed boxes and arrowheads) following in situ drift correction. Scale bars, 500 nm (**a**, **b**), 100 nm (**c**).

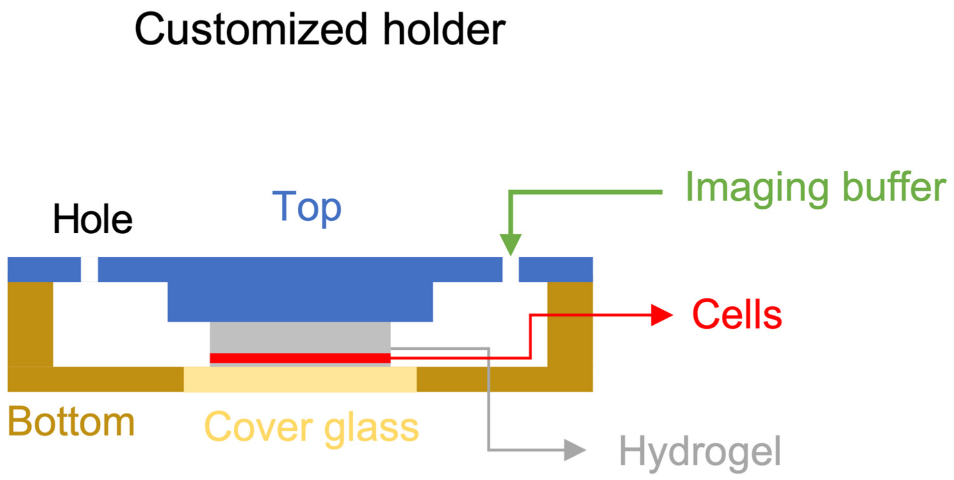

Supplementary Figure 4. Schematic diagram of the customized sample holder for Ex-dSTORM imaging. By sandwiching the hydrogel after re-embedding, we can minimize lateral drift without chemical modification of the cover glass. Two small holes on both sides of the top panel are the inlet and outlet for imaging buffer replacement. The nearly closed system could ensure the least oxidation of imaging buffers arising from the air.

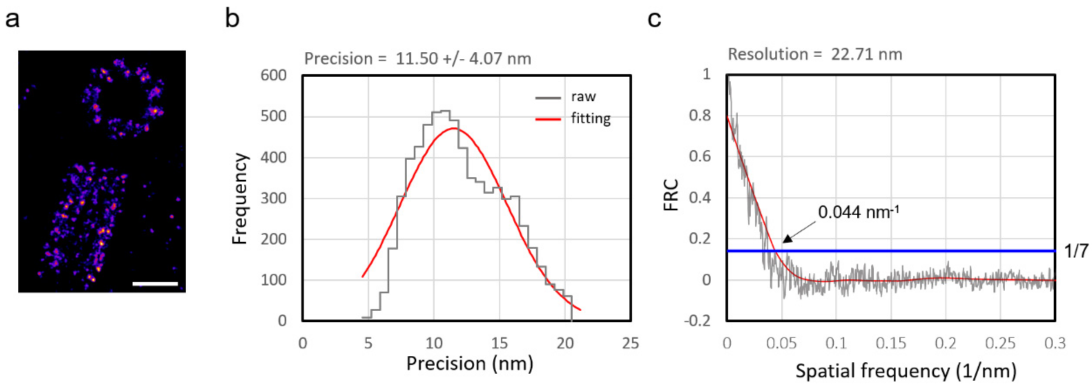

d

	Localization precision (nm)	Resolution (nm)
Original scale	11.50 ± 4.07	22.71
Scaled by 1/3.92*	-	5.79 (shortest distance between two labeled molecules that can be distinguished with Ex-dSTORM)

* 3.92 is the averaged expansion factor in this work.

Supplementary Figure 5. Validation of our Ex-dSTORM resolution. a Representative Ex-dSTORM image of Ac-Tub in RPE-1 cell for the resolution analysis. **b** Histograms analysis of localization precision from single molecules per switching event. The mean localization precision of the image is equal to 11.5 nm. **c** FRC analysis on the image shown in **a** with a threshold designated to 1/7, as indicated in the blue line. The FRC cutoff returns 0.044 nm^{-1}, and the resolution, the reciprocal of the FRC cutoff, equals 22.71 nm. **d** Table of resolution data from **b** and **c** along with an additional row of effective resolution value incorporated with the expansion factor gained in **Supplementary Table 1**. The shortest distance between two labeled molecules that can be distinguished with Ex-dSTORM is thus equal to 5.79 nm. Scale bar, 500 nm (**a**). Source data are provided as a Source Data file.

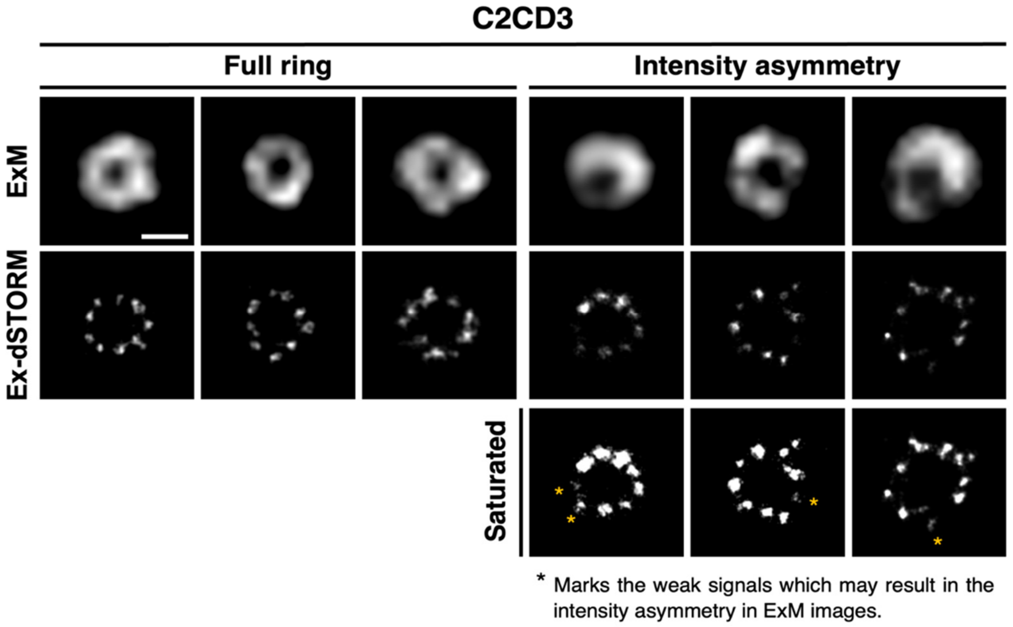

Supplementary Figure 6. Intensity Asymmetry of C2CD3. Imaging results of C2CD3 were categorized into the full ring and intensity asymmetry parts by distinct morphologies displayed in the ExM images. Following the group of intensity asymmetry, the Ex-dSTORM images were further processed to allow the single-molecule signals to become saturated and lead to the revelation of low-intensity puncta. Asterisks denote the weak signals that may result in intensity asymmetry. Scale bar, 100 nm.

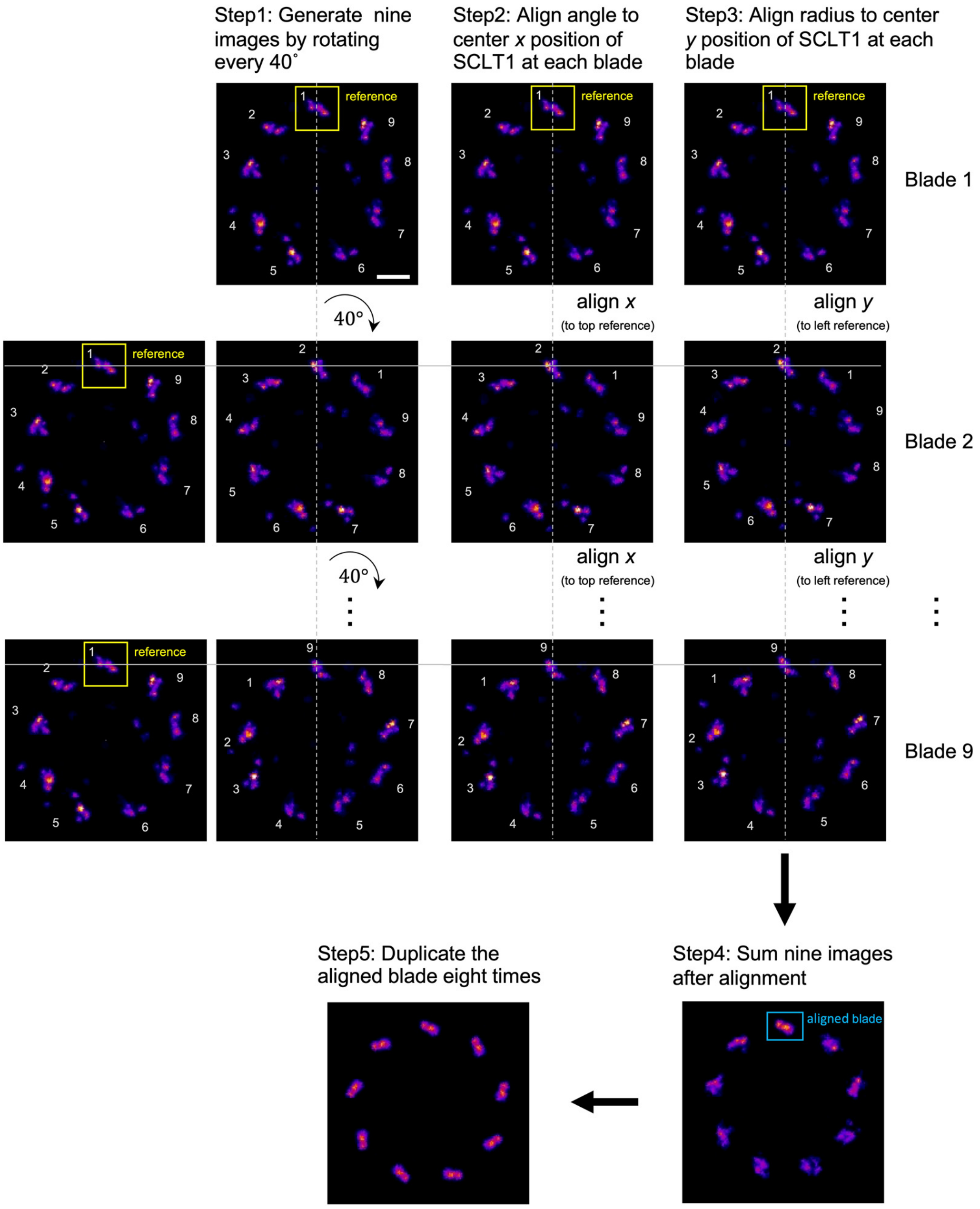

Supplementary Figure 7. Procedure of averaging the molecular feature of DAP in Ex-dSTORM image (refer to **Fig. 2e**). To strengthen the ultra-detailed features of the proteins, we average the Ex-dSTORM signals with representative images. First, we rotate the original Ex-dSTORM image every 360˚/9 around its geometrical center (9 images). Second, we align the angle to the center *x* position of SCLT1 at each blade. Third, we perform the longitudinal movement of every image to align the center of the blade compared with the reference blade. Fourth, we combine nine aligned images to extract the features of individual proteins (top aligned blade). Finally, we duplicate the aligned blade from step 4 eight times with a 40˚ interval to acquire the final averaged image with ultra-detailed features. Scale bar, 100 nm.

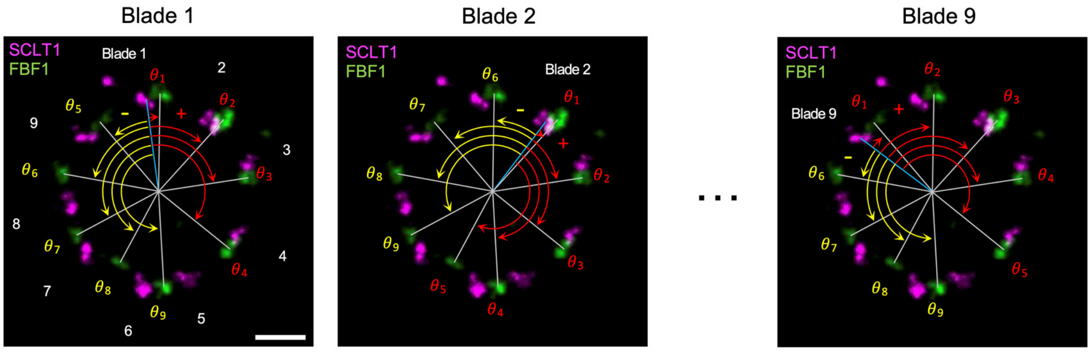

Supplementary Figure 8. Measurement of relative angles. We apply the angular analysis to determine the relative radial direction among proteins (observed from the distal view of the centriole). The angular positions of each protein punctum are first recorded in the Ex-dSTORM images. The difference in angles is then determined from the center of reference (blue line) to the center of targets (gray lines) and assigned with the direction (minus sign: counterclockwise, plus sign: clockwise). Angle differences for a single reference are obtained by calculating the included angles between the reference (blue line) and the targets (gray lines) from the nearest target signals (θ_1) to the farthest signals (θ_4 and θ_9). The rest of the angle differences for other references in distinct blades are measured in the same manner (Blade1 to Blade 9). Scale bar, 100 nm.

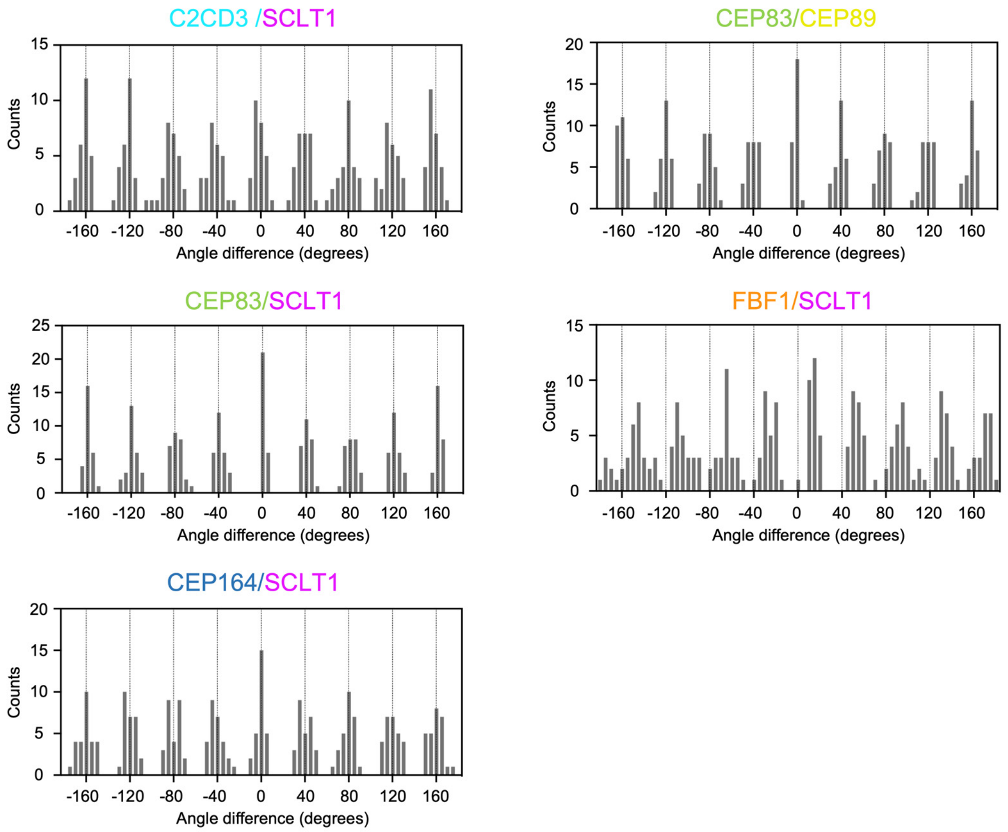

Supplementary Figure 9. Angular analyses in the radial direction revealing the protein-protein angular relationship. The histogram analysis of angle differences of various protein pairs reveals the angular spacing and the relative angle (n = 243 measured points from 3 centrioles for each pair). In all protein pairs, the angular spacings between blades are found to be multiples of 40˚. For all pairs except the FBF1-SCLT1 pair, the relative angle between blades is the multiples of 40˚, indicating these proteins are located in the same radial direction in a single blade. As for the FBF1-SCLT1 pair, the result demonstrates that FBF1 is positioned in the distinct radial direction against SCLT1 and other DAP proteins. Source data are provided as a Source Data file.

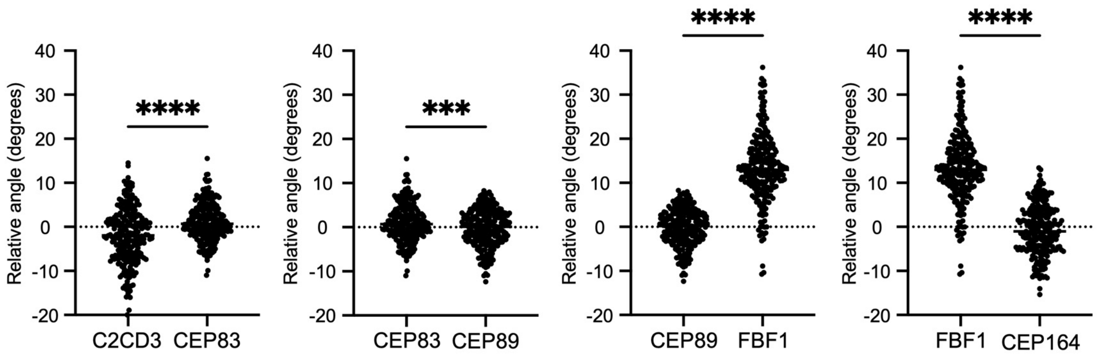

Supplementary Figure 10. Statistical analysis of the relative angle among DAP proteins and C2CD3. The relative angles between DAP proteins and SCLT1 are demonstrated in the above plots. The statistical analyses reveal the statistical significance of the relative angles among different DAP proteins (n = 243 measured points from 3 cenrioles for each data set; exact p values: 1.364×10^{-9} (C2CD3-CEP83), 0.001 (CEP9-CEP89), $< 10^{-15}$ (CEP89-FBF1), and $< 10^{-15}$ (FBF1-CEP164), ***$p<0.001$, ****$p<0.0001$, unpaired two-tailed *t*-test). The differences in relative angles are significant due to a more discriminative imaging resolution with Ex-dSTORM. Source data are provided as a Source Data file.

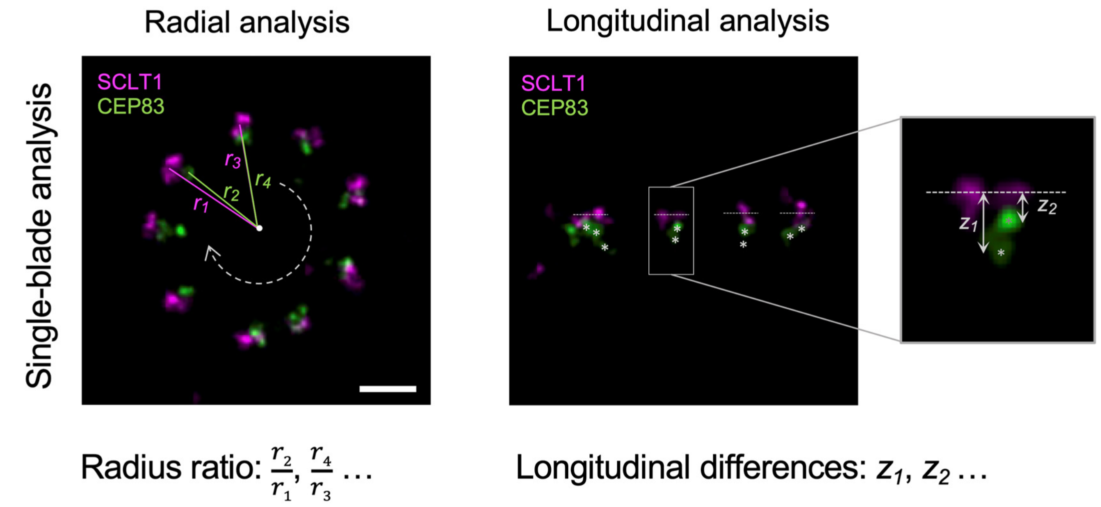

Supplementary Figure 11. Single-blade resolution for radial and longitudinal analyses. In radial analysis, we calculated the radius ratio, not an absolute value, to eliminate the possible blade-to-blade and cell-to-cell variation resulting from different sample batches after re-embedding. The radius ratios are calculated in a single blade by dividing the radius of target signals (CEP83) against the radius of reference signals (SCLT1), and the same processes are repeated in all the blades. In longitudinal analysis, the longitudinal differences are obtained by measuring the longitudinal distance from the reference (dashed lines) to every punctum of targets (asterisks) in a single blade (z_1, z_2, inset). To minimize sample variations in the longitudinal analysis, we picked the data for the mean diameter of SCLT1 in every sample batch within a 5% difference. Scale bar, 100 nm.

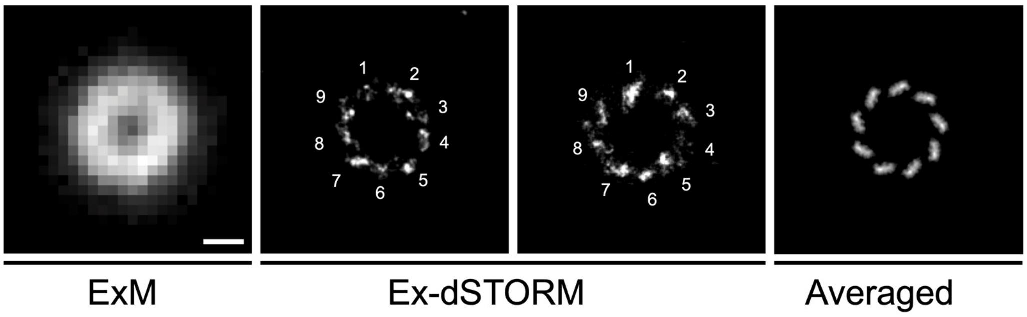

Supplementary Figure 12. Ex-dSTORM image revealing nine discrete microtubule triplets. With the spatial resolution of Ex-dSTORM, we can visualize the nine microtubule triples from the RPE-1 centrioles stained for Ac-tub. The averaged result from nine images rotated every 40˚ enhances the features of microtubule triplets with a specific chirality. Scale bar, 100 nm.

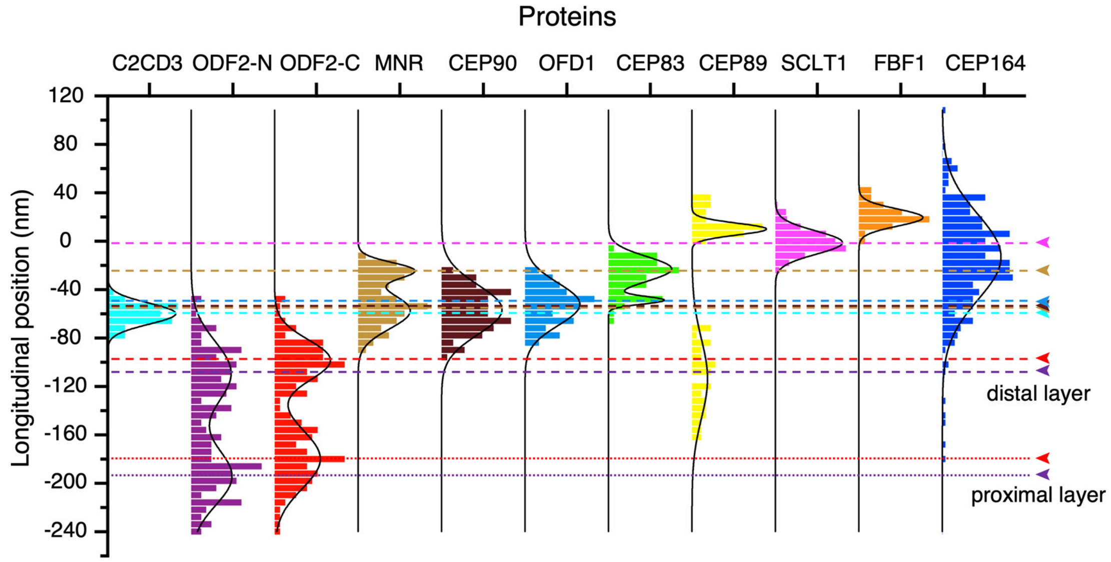

Supplementary Figure 13. Histogram analysis of the longitudinal positions of DAP proteins, C2CD3, MNR, CEP90, OFD1, and ODF2-C/N relative to SCLT1. Arrowheads marked the mean longitudinal position of SCLT1, MNR, OFD1, CEP90, ODF2-C, and ODF2-N (n = 7, 5, 6, 5, 6, 7, 9, 14, 24, 13 and centrioles for C2CD3, ODF2-N, ODF2-C, MNR, CEP90, OFD1, CEP83, CEP89, SCLT1, FBF1, and CEP164, respectively). MNR, CEP90, and OFD1 represent a broader distribution than SCLT1. Specifically, the two-layered distribution of MNR can be observed. Similarly, the two-layered pattern of ODF2-C/N can be identified in the analysis; one is located near DAPs, and the other is situated in the sDAPs. The distal ODF2-C/N is localized lower to other core DAP proteins, suggesting that this layer of ODF2 may be involved in the pedestal structure of the DAP. Source data are provided as a Source Data file.

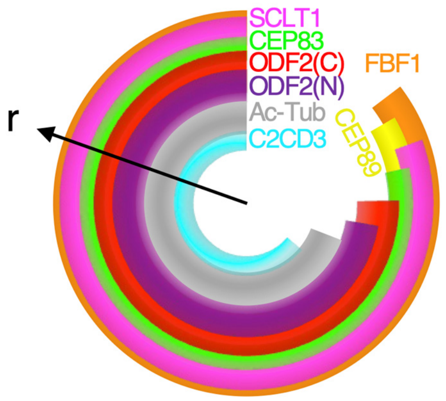

Supplementary Figure 14. ODF2-C/N localizing to the gap between CEP83 and the centriole wall. The radial coverage of DAP proteins, ODF2-C/N, MT triplets, and C2CD3 (mean $\pm$ 3 SD for each protein) are illustrated. ODF2-C and ODF2-N are recognized in the gap with a smaller radius than the outer DAP.

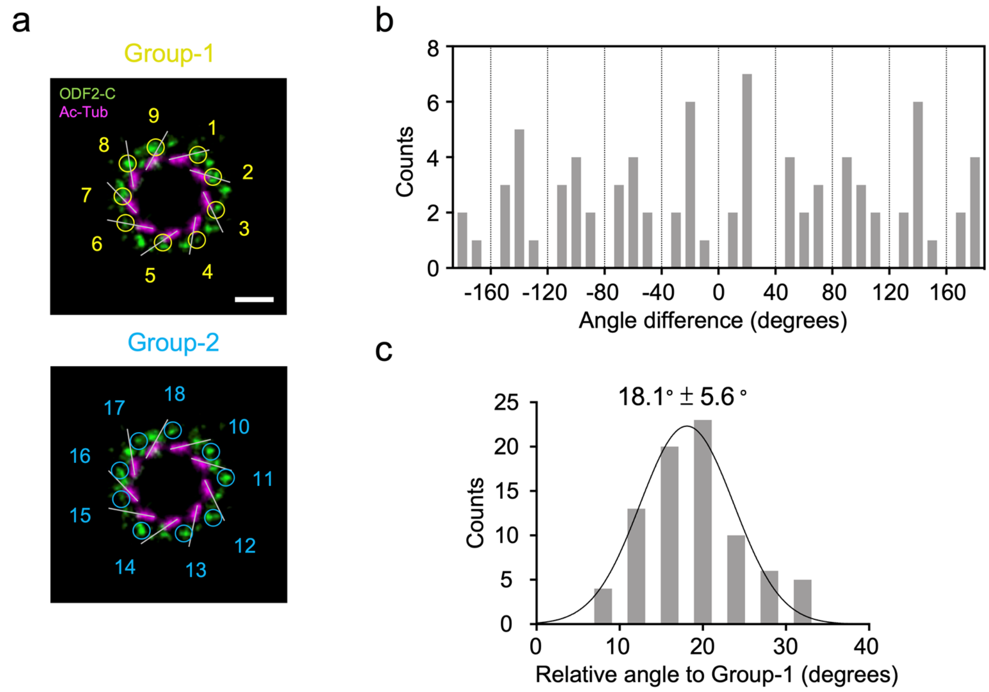

Supplementary Figure 15. Quantification of the angular offset between two groups of ODF2. a Representative image of two groups of ODF2 with Ac-Tub as the reference. **b** Histogram analysis of the relative angle between group-1 and group-2 of ODF2, utilizing group-1 of ODF2 as the reference and the analytical method described in **Supplementary Figures 8** and **9** (n = 81 measured points from one image). **c** Gaussian fitting with all measured points revealing an 18° clockwise offset of group-2 of ODF2 relative to group-1 of ODF2. Scale bar, 100 nm (**a**). Source data are provided as a Source Data file.

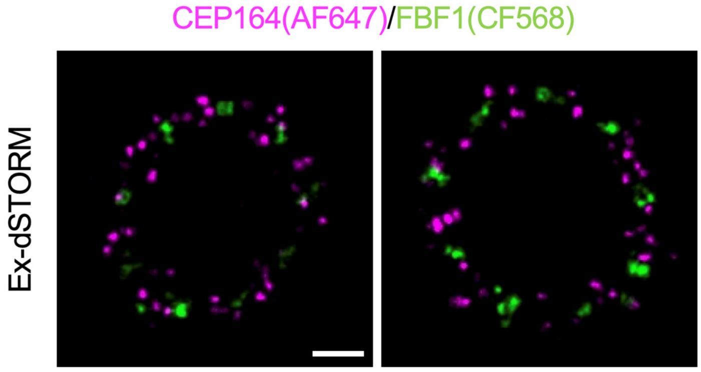

Supplementary Figure 16. Representative Ex-dSTORM images showing negligible crosstalk using AF647. Images of protein pair CEP164 (AF647) and FBF1 (CF568) with distinct angular distributions along circumferential direction demonstrate negligible crosstalk between two imaging channels when AF647 is used for staining the protein with similar or less copy number compared to another in CF568. Scale bar, 100nm.

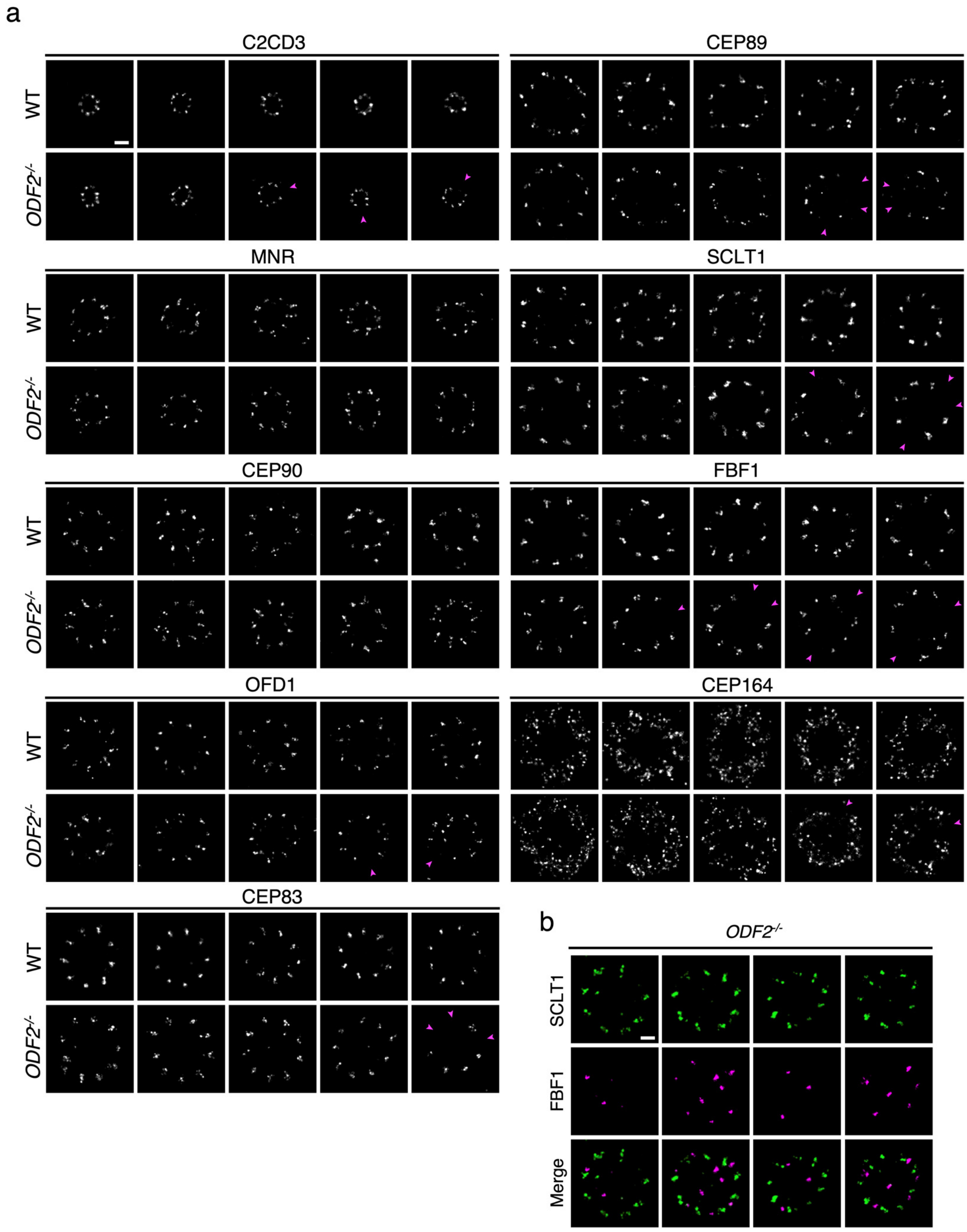

Supplementary Figure 17. Effect of *ODF2* depletion on the structural arrangement of C2CD3 and DAP proteins. a Representative Ex-dSTORM images of C2CD3 and DAP proteins in WT and *ODF2*$^{-/-}$ cells. The spatial organization of symbolic nine-fold symmetric patterns could be observed in the WT images. Nevertheless, for the *ODF2*$^{-/-}$ centrioles, some non-ninefold symmetric patterns are revealed where the missing puncta are indicated with arrowheads (magenta). **b** Representative two-color Ex-dSTORM images of FBF1 and SCLT1 in *ODF2*$^{-/-}$ cells. FBF1 is grossly impacted upon *ODF2* depletion, while the typical SCLT1 pattern is mostly retained. Scale bars, 100 nm (**a**, **b**).

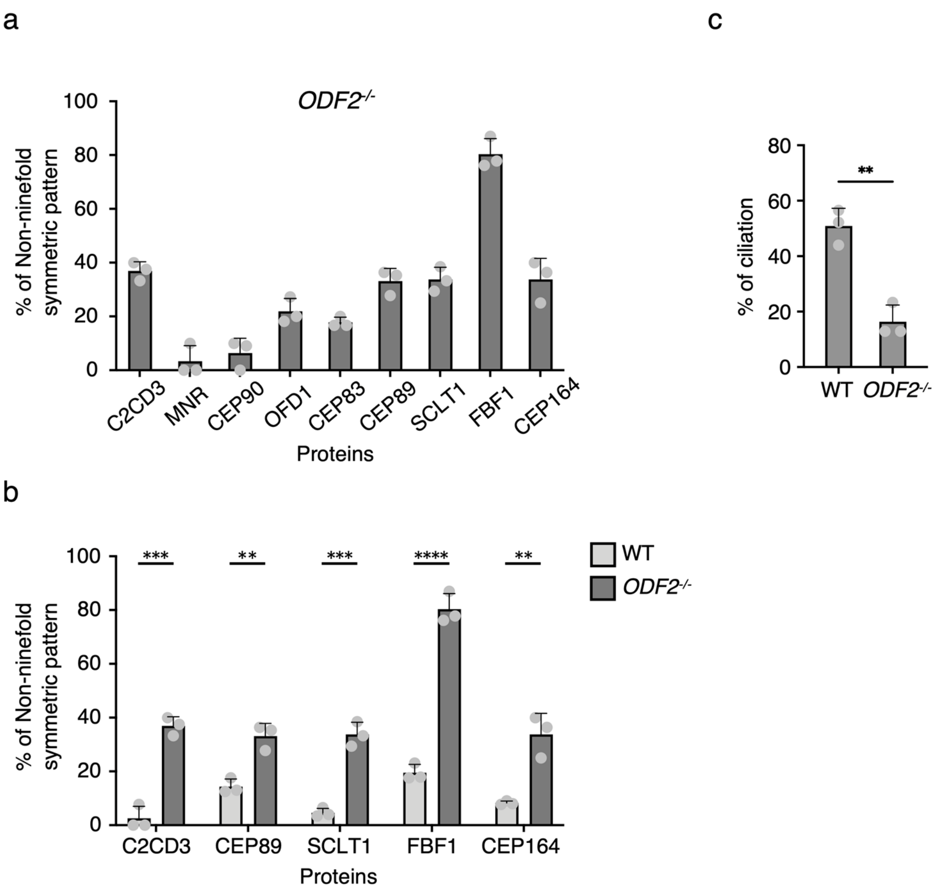

Supplementary Figure 18. Quantitative analyses of disorganized DAP proteins upon *ODF2* depletion. a Percentage of non-ninefold symmetric distribution of all proteins recorded in *ODF2*$^{-/-}$ cells (n = 3 independent experiments; at least five centrioles were measured per experiment for C2CD3; at least eight centrioles were measured per experiment for other proteins). **b** Comparison of non-ninefold symmetric pattern of five selected proteins from **a** with the fraction over 1/3, compared across the WT and *ODF2*$^{-/-}$ cells (n = 3 independent experiments; at least five centrioles were measured per experiment for C2CD3; at least eight centrioles were measured per experiment for other proteins). An interesting remark is given to FBF1, which shows the most considerable fraction of irregular patterns among all other proteins (p values: 0.0004399 (C2CD3), 0.003968 (CEP89), 0.0004851 (SCLT1), 0.0000901 (FBF1), 0.004919 (CEP164)). **c** Comparison of ciliation percentage between WT and *ODF2*$^{-/-}$ cells (n = 3 independent experiments for both WT and *ODF2*$^{-/-}$ cells; p value = 0.002406). All data are presented as mean $\pm$ SD. Statistical analyses were performed based on two-tailed unpaired t-test individually (**p<0.01, ***p<0.001, ****p<0.0001). Source data are provided as a Source Data file.

Supplementary Table 1 Determination of the expansion factor via dividing the mean diameter of SCLT1 analyzed from Ex-dSTORM images by that from dSTORM images

SCLT1	Diameter (mean ± sd, nm)	Expansion factor
dSTORM	402.0 ± 20.7 (12 MCs)	–
Ex-dSTORM	1575.1 ± 128.0 (126 BLs)	1575.1/402.0 = 3.92

(MC = mother centriole, BL = blade)

Supplementary Table 2 Ultra-detailed analyses of core DAP proteins

Proteins	Characteristic length (XY) (mean ± sd, nm)	Rotation angle (mean ± sd, degrees)	Longitudinal distance (Z) (mean ± sd, nm)	Inclination angle (mean ± sd, degrees) *
C2CD3	–	broad distribution (31BLs)	–	–
CEP83	20.2 ± 3.3 (58 BLs)	30.4 ± 11.6° (45 BLs)	25.9 ± 5.5 (36 BLs)	52.1 ± 19.0°
CEP89	29.3 ± 5.5 (32 BLs)	51.3 ± 10.9° (38 BLs)	11.2 ± 5.0 (15 BLs)	21.0 ± 9.7°
SCLT1	31.4 ± 5.5 (55 BLs)	55.5 ± 13.0° (41 BLs)	8.6 ± 3.9 (25 BLs)	15.3 ± 7.6°
FBF1	30.0 ± 5.5 (49 BLs)	broad distribution (43 BLs)	10.6 ± 4.6 (20 BLs)	19.5 ± 10.1°

(BL = blade)

*Inclination angles were calculated as the ratio of characteristic length to longitudinal distance.

Supplementary Table 3 Ultra-resolved diameter, relative angle, and chirality of various proteins

Proteins	Diameter (mean ± sd, nm)	Relative angle (mean ± sd, degrees)	Chirality
C2CD3	146.4 ± 14.3 (36 BLs)	-1.7 ± 6.3° (3 MCs)	–
MNR	237.4 ± 8.9 (63 BLs)	13.6 ± 4.2°(4 MCs)	clockwise **
CEP90	272.0 ± 8.7 (54 BLs)	10.9 ± 2.5°(5 MCs)	counterclockwise **
distal ODF2-N	273.5 ± 20.2 (45 BLs)	15.3 ± 4.2° (6 MCs)	–
distal ODF2-C	307.0 ± 19.0 (36 BLs)	1.8 ± 5.5° (5 MCs)	clockwise **
OFD1	322.6 ± 10.0 (54 BLs)	7.4 ± 2.0°(4 MCs)	counterclockwise**
CEP83	359.4 ± 14.5 (36 BLs)	0.6 ± 4.0° (3 MCs)	counterclockwise
CEP89	366.1 ± 19.4 (36 BLs)	0.2 ± 4.5° (3 MCs)	counterclockwise
SCLT1	401.8 ± 32.6 (126 BLs)	0°	counterclockwise
FBF1	408.2 ± 25.8 (34 BLs)	12.9 ± 5.3° (3 MCs)	–
CEP164	417.5 ± 76.3 (36 BLs) *	-0.9 ± 6.2° (3 MCs)	clockwise
Ac-Tub	200.4 ± 14.6 (42 BLs)	18.8 ± 7.0° (4 MCs)	clockwise

(BL = blade, MC= mother centriole)

*Radial distribution of CEP164: 265.1 – 570.3 nm (broad distribution denoted as mean ± 2 sd)

** 54% of MNR represents counterclockwise chirality (39/72 BLs)

63% of CEP90 represents counterclockwise chirality (57/90 BLs)

44% of ODF2-C represents counterclockwise chirality (48/108 BLs)

67% of OFD1 represents counterclockwise chirality (48/72 BLs)

Supplementary Table 4 Ultra-resolved longitudinal locations of various proteins

Proteins	Longitudinal position relative to SCLT1 (mean ± sd, nm)	Number of measured points
C2CD3	-60.0 ± 8.7	42 (7 MCs)
MNR	-24.2 ± 6.6; -57.6 ± 14.9	61 (5 MCs)
CEP90	-56.7 ± 20.4	85 (6 MCs)
ODF2-N	-109.1 ± 26.0; -194.5 ± 23.6	205 (6 MCs)
ODF2-C	-98.3 ± 16.8; -180.0 ± 24.4	161(6 MCs)
OFD1	-52.2 ± 17.5	56 (7 MCs)
CEP83	-23.0 ± 10.3; -49.0 ± 3.2	64 (9 MCs)
CEP89	9.9 ± 4.9; -114 ± 33.3	86 (14 MCs)
SCLT1	-1.3 ± 9.6	265 (24 MCs)
FBF1	19.4 ± 7.0	68 (13 MCs)
CEP164	-15.9 ± 40.0*	289 (9 MCs)

(MC = mother centriole)

*Longitudinal distribution of CEP164: -95.9 – 64.1nm (broad distribution denoted as mean ± 2 sd)

Supplementary Table 5 List of primary antibodies in the study

Designation of antibody	Host	Source or reference	Immunogen (Sequence)	Identifiers	Dilution (Ex-dSTORM)
C2CD3	rabbit IgG	Sigma-Aldrich	human C2CD3 (803-905 aa)	HPA040433	1/250
Acetyl-alpha Tubulin (Ac-Tub, 6-11-b-1)	mouse IgG	Thermo Fisher	–	32-2700	1/200
CEP83	rabbit IgG	Sigma-Aldrich	human CEP83 (578-677 aa)	HPA038161	1/100
CEP89	rat IgG	Tanos et al. Genes & development (2013)[3]	–	–	1/100
SCLT1	rat IgG	Tanos et al. Genes & development (2013)[3]	–	–	1/100
FBF1	rabbit IgG	Proteintech	human FBF1 (20-347 aa)	11531-1-AP	1/100
CEP164	rabbit IgG	Proteintech	human CEP164 (1-112 aa)	22227-1-AP	1/250
ODF2-C	rabbit IgG	Abcam	–	ab43840	1/100
ODF2-N	rabbit IgG	Sigma-Aldrich	human ODF2 (39-200 aa)	HPA001874	1/100
MNR (KIAA0753)	rabbit IgG	Novus Biologicals	human CEP90 (658-761 aa)	NBP1-90929	1/100
CEP90 (PIBF1)	rabbit IgG	Proteintech	human CEP90 (452-757 aa)	14413-1-AP	1/100
OFD1	rabbit IgG	Sigma-Aldrich	human CEP90 (439-530 aa)	HPA031103	1/100
ATP synthase	mouse IgG	Abcam	–	ab109867	1/125
CEP164	goat IgG	Santa Cruz Biotechnology	human CEP164 N-terminus	sc-240226	1/100
Polyglutamylated tubulin (GT335)	mouse IgG	AdipoGen	–	AG-20B0020-C100	1/200

(aa = amino acid)

Supplementary references

1. Gaudin N*, et al.* Evolutionary conservation of centriole rotational asymmetry in the human centrosome. *Elife* **11**, e72382 (2022).
2. Yang TT*, et al.* Super-resolution architecture of mammalian centriole distal appendages reveals distinct blade and matrix functional components. *Nature communications* **9**, 1-11 (2018).
3. Tanos BE*, et al.* Centriole distal appendages promote membrane docking, leading to cilia initiation. *Genes & development* **27**, 163-168 (2013).
