# Supplementary material for: Single-molecule localization microscopy reveals the ultrastructural constitution of distal appendages in expanded mammalian centrioles
Source: Nat Commun. 2023 Mar 27;14:1688. doi: 10.1038/s41467-023-37342-x (PMC10043031; doi:10.1038/s41467-023-37342-x)
Supplement: Supplementary file 3 — Reporting Summary [file 41467_2023_37342_MOESM3_ESM.pdf]

nature portfolio

Corresponding author(s): T. Tony Yang
Last updated by author(s): Feb 15, 2023

Reporting Summary

Nature Portfolio wishes to improve the reproducibility of the work that we publish. This form provides structure for consistency and transparency in reporting. For further information on Nature Portfolio policies, see our Editorial Policies and the Editorial Policy Checklist.

Statistics

For all statistical analyses, confirm that the following items are present in the figure legend, table legend, main text, or Methods section.

n/a	Confirmed	
☐	☒	The exact sample size (*n*) for each experimental group/condition, given as a discrete number and unit of measurement
☐	☒	A statement on whether measurements were taken from distinct samples or whether the same sample was measured repeatedly
☐	☒	The statistical test(s) used AND whether they are one- or two-sided<br>*Only common tests should be described solely by name; describe more complex techniques in the Methods section.*
☒	☐	A description of all covariates tested
☒	☐	A description of any assumptions or corrections, such as tests of normality and adjustment for multiple comparisons
☐	☒	A full description of the statistical parameters including central tendency (e.g. means) or other basic estimates (e.g. regression coefficient) AND variation (e.g. standard deviation) or associated estimates of uncertainty (e.g. confidence intervals)
☐	☒	For null hypothesis testing, the test statistic (e.g. *F*, *t*, *r*) with confidence intervals, effect sizes, degrees of freedom and *P* value noted<br>*Give P values as exact values whenever suitable.*
☒	☐	For Bayesian analysis, information on the choice of priors and Markov chain Monte Carlo settings
☒	☐	For hierarchical and complex designs, identification of the appropriate level for tests and full reporting of outcomes
☒	☐	Estimates of effect sizes (e.g. Cohen's *d*, Pearson's *r*), indicating how they were calculated

Our web collection on statistics for biologists contains articles on many of the points above.

Software and code

Policy information about availability of computer code

Data collection	Ex-dSTORM and conventional widefield images were collected with a custom-built setup based on a commercial inverted microscope (Eclipse Ti-E, Nikon) detailed in the manuscript (Methods section). The microscopy system, mainly consisting of a microscope body, laser module, and camera, is integrated by MetaMorph (version 7.8.8.0, Molecular Devices). Superresolution dSTORM imaging was rendered with the Superresolution Module (Wavetracer, version 1.50, Molecular Devices).
Data analysis	Ex-dSTORM data were processed using a homemade code based on Labview (version 14.0.1), Matlab (version R2021b), and ImageJ (version 1.52) for in-situ drift correction. The position of the individual single-molecule peak was then localized using MetaMorph Superresolution Module (version 7.8.8.0, Molecular Devices) based on a wavelet segmentation algorithm (version 1.50, Molecular Devices). The superresolution images were cleaned with the gaussian filter using ImageJ (version 1.52 p). The quantitative analyses were performed by ImageJ and Prism (version 9.4.1. GraphPad). The 3D computation model in the study was built by Blender (version 2.93.4). The FRC analysis was conducted by ImageJ plugin GDSC SMLM.

For manuscripts utilizing custom algorithms or software that are central to the research but not yet described in published literature, software must be made available to editors and reviewers. We strongly encourage code deposition in a community repository (e.g. GitHub). See the Nature Portfolio guidelines for submitting code & software for further information.

Data

Policy information about availability of data

All manuscripts must include a data availability statement. This statement should provide the following information, where applicable:

- Accession codes, unique identifiers, or web links for publicly available datasets
- A description of any restrictions on data availability
- For clinical datasets or third party data, please ensure that the statement adheres to our policy

All the data supporting the findings described in this study are available within the article and its supplementary information, and Source Data file.

Human research participants

Policy information about studies involving human research participants and Sex and Gender in Research.

Reporting on sex and gender	N/A
Population characteristics	N/A
Recruitment	N/A
Ethics oversight	N/A

Note that full information on the approval of the study protocol must also be provided in the manuscript.

Field-specific reporting

Please select the one below that is the best fit for your research. If you are not sure, read the appropriate sections before making your selection.

☒ Life sciences ☐ Behavioural & social sciences ☐ Ecological, evolutionary & environmental sciences

For a reference copy of the document with all sections, see nature.com/documents/nr-reporting-summary-flat.pdf

Life sciences study design

All studies must disclose on these points even when the disclosure is negative.

Sample size	There was no predetermined sample size. The sample size was selected based on our prior literature report (Yang, T. Tony, et al. Nature communications 9.1 (2018): 1-11.), which could sufficiently demonstrate the availability of our analyses. A minimum of five Ex-dSTORM images were captured for quantitative analyses at each condition or each data set.
Data exclusions	Data from failed experiments, including failed in-situ drift correction and wrong observed orientation, were excluded. The criteria of exclusion were predetermined.
Replication	In all cases, the replication was successful. The number of replications were provided in the figure legends and supplementary tables.
Randomization	Only centrioles with the top-view and lateral-view orientations were imaged. The step of searching for cells is randomly performed throughout all the experiments.
Blinding	Blind counting was utilized when the quantitative analysis for specific experiments was performed, for example, ciliation rate and number of non-ninefold symmetric pattern of distal appendages proteins.

Reporting for specific materials, systems and methods

We require information from authors about some types of materials, experimental systems and methods used in many studies. Here, indicate whether each material, system or method listed is relevant to your study. If you are not sure if a list item applies to your research, read the appropriate section before selecting a response.

Materials & experimental systems

n/a	Involved in the study
☐	☒ Antibodies
☐	☒ Eukaryotic cell lines
☒	☐ Palaeontology and archaeology
☒	☐ Animals and other organisms
☒	☐ Clinical data
☒	☐ Dual use research of concern

Methods

n/a	Involved in the study
☒	☐ ChIP-seq
☒	☐ Flow cytometry
☒	☐ MRI-based neuroimaging

Antibodies

Antibodies used

Primary antibodies:
-rabbit polyclonal anti-C2CD3 (1/250, HPA040433, Sigma-Aldrich)
-mouse monoclonal anti-Acetyl-alpha Tubulin (Ac-Tub) (1/200, 32-2700, Thermo Fisher)
-rabbit polyclonal anti-CEP83 (1/100, HPA038161, Sigma-Aldrich)
-rat polyclonal anti-CEP89 (1/100, customized by Meng-Fu Bryan Tsou)
-rat polyclonal anti-SCLT1 (1/100, customized by Meng-Fu Bryan Tsou)
-rabbit polyclonal anti-FBF1 (1/100, 11531-1-AP, Proteintech)
-rabbit polyclonal anti-CEP164 (1/250, 22227-1-AP, Proteintech)
-rabbit polyclonal anti-ODF2-C (1/100, ab43840, Abcam)
-rabbit polyclonal anti-ODF2-N (1/100, HPA001874, Sigma-Aldrich)
-rabbit polyclonal anti-MNR (KIAA0753) (1/100, NBP1-90929, Novus Biologicals)
-rabbit polyclonal anti-CEP90 (PIBF1) (1/100, 14413-1-AP, Proteintech)
-rabbit polyclonal anti-OFD1 (1/100, HPA031103, Sigma-Aldrich)
-mouse monoclonal anti-ATP synthase (1/125, ab109867, Abcam)
-goat polyclonal anti-CEP164 (1/100, sc-240226, Santa Cruz Biotechnology)
-mouse monoclonal anti-Polyglutamylated tubulin (GT335) (1/200, AG-20B0020-C100, AdipoGen)

Secondary antibodies:
-donkey anti-mouse Alexa Fluor 647 IgG (H+L), (1/100, A31571, Thermo Fisher)
-donkey anti-rabbit Alexa Fluor 647 IgG (H+L), (1/100, A31573, Thermo Fisher)
-donkey anti-rat Alexa Fluor 647 IgG (H+L), (1/100, A21247, Thermo Fisher)
-donkey anti-goat Alexa Fluor 647 IgG (H+L), (1/100, A-21447, Thermo Fisher)
-goat anti-mouse Atto 647N IgG (H+L), (1/100, 50185-1ML-F, Sigma-Aldrich)
-donkey anti-mouse CF568 IgG (H+L), (1/100, 20105, Biotium)
-donkey anti-rabbit CF568 IgG (H+L), (1/100, 20098, Biotium)
-donkey anti-rat CF568 IgG (H+L), (1/100, 20092, Biotium)
-donkey anti-mouse Alexa Fluor 488 IgG (H+L), (1/100, A21202, Thermo Fisher)

Self conjugated secondary antibodies:
Dyomics 654 (Dy654)-conjugated secondary antibodies were custom-made by conjugating Dy654 N-hydroxysuccinimidyl (NHS) ester (654-01; Dyomics) to different IgG antibodies respectively (anti-mouse 715-005-151, anti-rabbit 711-005-152, anti-rat 712-005-153; Jackson ImmunoResearch).

Validation

All the antibodies used here are validated by the vendors indicated above. Most of them have been extensively used in the past by us and others in many references. Below we list one reference for each antibody, or the information regarding validation and application in the manufacturer's website.

Primary antibodies:
rabbit polyclonal anti-C2CD3 (1/250, HPA040433, Sigma-Aldrich) - https://www.atlasantibodies.com/products/antibodies/primary-antibodies/triple-a-polyclonals/c2cd3-antibody-hpa040433/
-mouse monoclonal anti-Acetyl-alpha Tubulin (Ac-Tub) (1/200, 32-2700, Thermo Fisher) - DOI: 10.1038/s41592-020-0859-z
-rabbit polyclonal anti-CEP83 (1/100, HPA038161, Sigma-Aldrich) - DOI: 10.1038/s41467-018-04469-1
-rat polyclonal anti-CEP89 (1/100, customized by Meng-Fu Bryan Tsou) - DOI: 10.1038/s41467-018-04469-1
-rat polyclonal anti-SCLT1 (1/100, customized by Meng-Fu Bryan Tsou) - DOI: 10.1038/s41467-018-04469-1
-rabbit polyclonal anti-FBF1 (1/100, 11531-1-AP, Proteintech) - DOI: 10.1038/s41467-018-04469-1
-rabbit polyclonal anti-CEP164 (1/250, 22227-1-AP, Proteintech) - DOI: 10.1038/s41467-018-08216-4
-rabbit polyclonal anti-ODF2-C (1/100, ab43840, Abcam) - DOI: 10.7554/eLife.53580
-rabbit polyclonal anti-ODF2-N (1/100, HPA001874, Sigma-Aldrich) - DOI: 10.7554/eLife.53580
-rabbit polyclonal anti-MNR (KIAA0753) (1/100, NBP1-90929, Novus Biologicals) - DOI: 10.1371/journal.pbio.3001782
-rabbit polyclonal anti-CEP90 (PIBF1) (1/100, 14413-1-AP, Proteintech) - DOI: 10.1371/journal.pbio.3001782
-rabbit polyclonal anti-OFD1 (1/100, HPA031103, Sigma-Aldrich) - DOI: 10.1371/journal.pbio.3001782
-mouse monoclonal anti-ATP synthase (1/125, ab109867, Abcam) - DOI: 10.1016/j.cell.2018.09.014
-goat polyclonal anti-CEP164 (1/100, sc-240226, Santa Cruz Biotechnology) - DOI: 10.1038/s41467-018-04469-1
-mouse monoclonal anti-Polyglutamylated tubulin (GT335) (1/200, AG-20B0020-C100, AdipoGen) - DOI: 10.1038/s41592-020-0859-z

Secondary antibodies:
-donkey anti-mouse Alexa Fluor 647 IgG (H+L), (1/100, A31571, Thermo Fisher) - DOI: 10.1016/j.str.2021.06.001
-donkey anti-rabbit Alexa Fluor 647 IgG (H+L), (1/100, A31573, Thermo Fisher) - DOI: 10.1016/j.str.2021.06.001
-donkey anti-rat Alexa Fluor 647 IgG (H+L), (1/100, A21247, Thermo Fisher) - DOI: 10.1016/j.str.2021.06.001

-donkey anti-goat Alexa Fluor 647 IgG (H+L), (1/100, A-21447, Thermo Fisher) - DOI: 10.1038/s41467-018-04469-1
-goat anti-mouse Atto 647N IgG (H+L), (1/100, 50185-1ML-F, Sigma-Aldrich) - DOI: 10.1073/pnas.1705623114
-donkey anti-mouse CF568 IgG (H+L), (1/100, 20105, Biotium) - DOI: 10.1038/s41467-022-29751-1
-donkey anti-rabbit CF568 IgG (H+L), (1/100, 20098, Biotium) - DOI: 10.1038/s41598-019-52397-x
-donkey anti-rat CF568 IgG (H+L), (1/100, 20092, Biotium) - DOI: 10.1083/jcb.202103003
-donkey anti-mouse Alexa Fluor 488 IgG (H+L), (1/100, A21202, Thermo Fisher) - DOI: 10.1038/s41467-018-04469-1

Self conjugated secondary antibodies:
Dyomics 654 (Dy654)-conjugated secondary antibodies were custom-made by conjugating Dy654 N-hydroxysuccinimidyl (NHS) ester (654-01; Dyomics) to different IgG antibodies respectively (anti-mouse 715-005-151, anti-rabbit 711-005-152, anti-rat 712-005-153; Jackson ImmunoResearch). - DOI: 10.1038/nmeth.1768

Eukaryotic cell lines

Policy information about cell lines and Sex and Gender in Research

Cell line source(s)	RPE-1 (WT) (purchased from ATCC): cell line provided by Dr. Jung-Chi Liao (Academia Sinica, Taiwan)<br>RPE-1 (CEP128 KO): cell line provided by Dr. Meng-Fu Bryan Tsou (Sloan Kettering Institute, New York)<br>RPE-1 (ODF2 KO): cell line provided by Dr. Meng-Fu Bryan Tsou (Sloan Kettering Institute, New York)
Authentication	Cell lines used were not further authenticated.
Mycoplasma contamination	Cell lines have been tested and are negative for mycoplasma contamination.
Commonly misidentified lines (See ICLAC register)	No commonly misidentified cell lines were used.
